# Statins and their impact on epigenetic regulation: insights into disease

**DOI:** 10.3389/fphar.2025.1621163

**Published:** 2025-07-17

**Authors:** Rafael Tamayo-Trujillo, Patricia Guevara-Ramírez, Santiago Cadena-Ullauri, Viviana A. Ruiz Pozo, Elius Paz-Cruz, Ana Karina Zambrano

**Affiliations:** Universidad UTE, Facultad de Ciencias de la Salud Eugenio Espejo, Centro de Investigación Genética y Genómica, Quito, Ecuador

**Keywords:** pharmacology, pharmacoepigenetics, epigenetics, statins, molecular biology, healthcare and well-being

## Abstract

Statins have been primarily used for the management of low-density lipoprotein cholesterol and cardiovascular diseases However, in recent years, research has identified potential applications beyond cholesterol regulation. Statins exhibit pleiotropic effects, due to their ability to modulate gene expression via epigenetic mechanisms, including DNA methylation, histone acetylation, and microRNA regulation. Clinical studies have correlated these epigenetic changes with various pathological conditions, such as inflammation, atherosclerosis, cancer, diabetes, and autoimmune disorders. Despite encouraging findings, further research is required to fully understand the molecular pathways associated with the epigenetic actions of statins and disease pathogenesis. This review describes the potential role of statins as epigenetic modulators and their relevance in human disease management.

## Introduction

Statins are the primary pharmacological approach for reducing elevated levels of low-density lipoprotein (LDL) cholesterol (Guadamuz et al., 2022). Their clinical importance is highlighted by their inclusion in the World Health Organization (WHO) Model List of Essential Medicines (EML) for the management of cardiovascular diseases (CVD) (Kishore et al., 2018).

The therapeutic effect of statins involves the inhibition of 3-hydroxy-3-methylglutaryl-CoA (HMG-CoA) reductase, a key regulatory enzyme in the cholesterol synthesis pathway. This enzyme catalyzes the conversion of HMG-CoA into L-mevalonate, a crucial precursor in endogenous cholesterol production (Sizar et al., 2024; Zaky et al., 2023). As a result of this, there is an increase in the upregulation of LDL receptors on cell surfaces, enhancing the uptake of circulating LDL cholesterol (Zaky et al., 2023). Given the strong association between elevated LDL cholesterol and CVDs (Zambrano et al., 2023; Jung et al., 2022), statins are widely recommended for the management and prevention of these diseases (Guadamuz et al., 2022).

Beyond the lipid-lowering properties of statins, they exert a range of pleiotropic effects by modulating the mevalonate pathway, thereby influencing various cellular processes (Kim et al., 2019). One key effect is their anti-inflammatory potential, which acts by reducing LDL cholesterol, an established contributor to systemic inflammation, statins indirectly reduce inflammatory responses (Yan et al., 2024). Additionally, statins can directly interfere with the production of pro-inflammatory cytokines such as interferon-gamma and tumor necrosis factor-alpha (TNF-α), thereby reducing immune system activation and inflammation. Statins have also been shown to reduce of C-reactive protein levels in human hepatocytes, further supporting its anti-inflammatory properties and suggesting liver-specific interactions (Kim et al., 2019).

Emerging evidence has also correlated statins with epigenetic modulation, including changes in DNA methylation, histone acetylation, and microRNA expression (Awosika et al., 2023). For instance, statins have been shown to inhibit the expression of histone deacetylases (HDAC) while enhancing histones H3 and H4 acetylation, promoting a transcriptionally active chromatin state (Awosika et al., 2023; Karlic et al., 2015; Feig et al., 2011; Singh et al., 2016; Tikoo et al., 2015). In addition, they may influence gene regulation by enhancing the expression of DNA methyltransferases (DNMTs) at promoter regions (Awosika et al., 2023; Karlic et al., 2015; Kodach et al., 2011).

The present review aims to describe the intricate interaction between statins and epigenetic mechanisms, emphasizing their broader implications in various disorders beyond cardiovascular disease.

## Epigenetics and statins: molecular mechanisms

**FIGURE 1:**
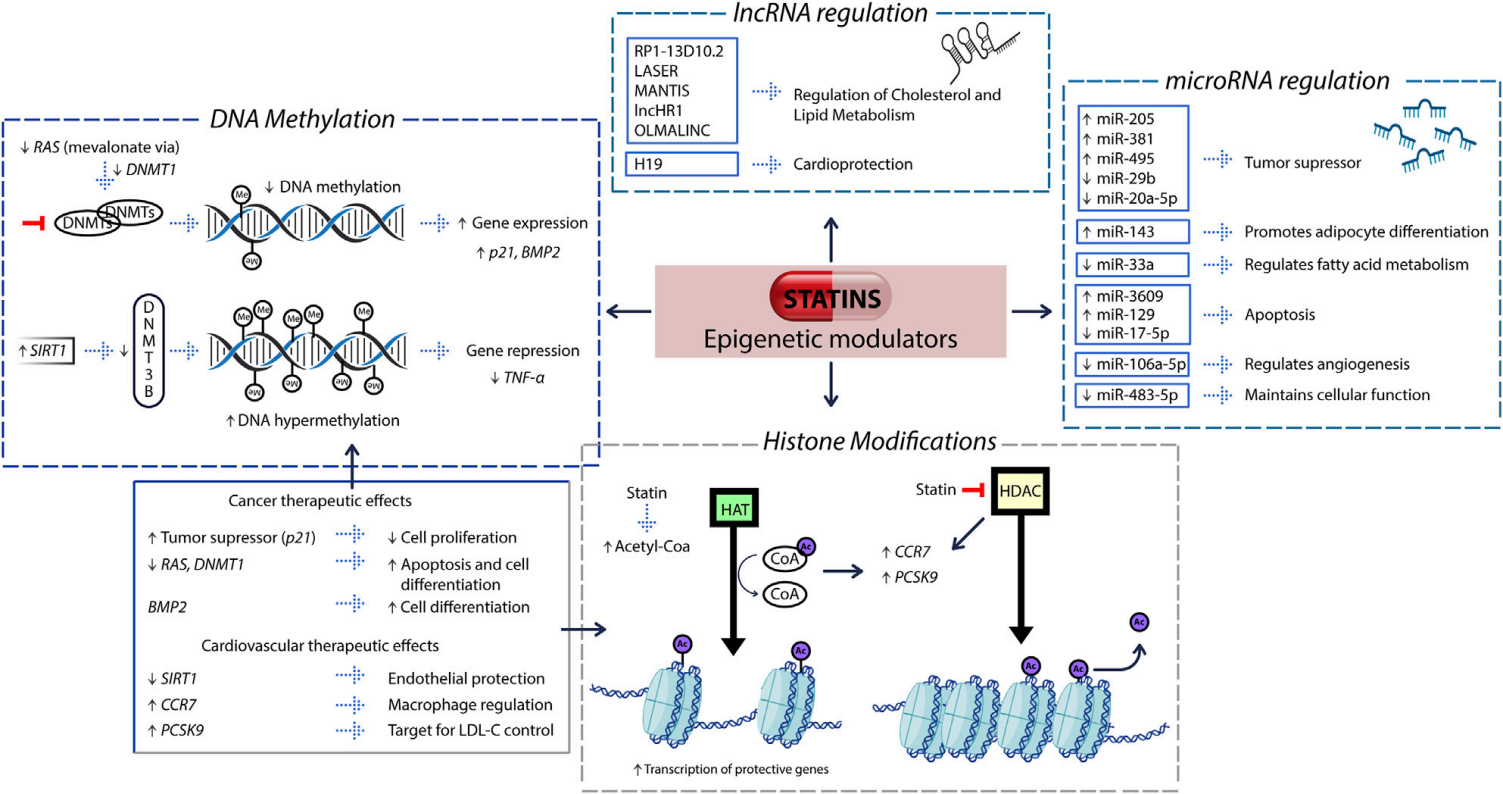
Epigenetic pathways modulated by statins and their functional implications. Statins modulate gene expression through four epigenetic mechanisms: (i) DNA methylation, (ii) histone modifications, (iii) microRNA regulation, and (iv) long non-coding RNA (lncRNA) regulation. (i) By inhibiting the mevalonate–RAS signaling pathway, statins downregulate DNA methyltransferase 1 (DNMT1), resulting in hypomethylation of promoter regions in tumor suppressor genes such as *p21* and *BMP2*. Concurrently, they upregulate *SIRT1*, which enhances the recruitment of DNMT3B, leading to hypermethylation and transcriptional silencing of pro-inflammatory genes such as TNF-α. These DNA methylation changes are represented in the figure as gray spheres labeled “Me.” (ii) Statins inhibit the activity of histone deacetylases (HDACs), increasing the intracellular availability of acetyl-CoA, a key substrate for histone acetyltransferases (HATs). HATs catalyze the addition of acetyl groups to histone tails, shown in the figure as purple spheres labeled “Ac,” resulting in chromatin relaxation and enhanced accessibility for transcription factors. This mechanism promotes the expression of genes such as *PCSK9* and *CCR7*, involved in cholesterol metabolism and immune cell trafficking, respectively. (iii) Statins modulate the expression of specific microRNAs, which regulate diverse cellular processes such as tumor suppression, lipid metabolism, apoptosis, and angiogenesis. (iv) Statins also influence the expression of long non-coding RNAs such as LASER, MANTIS, and H19, which play key roles in lipid homeostasis, vascular function, and cardiometabolic protection. These interconnected epigenetic pathways underline the multifaceted therapeutic potential of statins across oncology and cardiovascular health.

Statins act as epigenetic regulators through four main mechanisms: DNA methylation, histone modifications, microRNA expression, and long non-coding RNA regulation (Figure 1). These mechanisms contribute to their therapeutic impact in cardiovascular, metabolic, and inflammatory diseases.

### Statins and DNA methylation

Statins have been implicated in the regulation of epigenetic mechanisms, particularly by inhibiting DNMTs, which leads to reduced DNA methylation at gene promoter regions and subsequent activation of gene expression (Kayzuka et al., 2025). Statins can inhibit DNMT1 through two primary pathways. First, by blocking the mevalonate pathway, statins inhibit the isoprenylation of GTP-binding proteins, leading to the suppression of downstream signaling and reducing DNMT1 expression. Second, statins lower the production of interleukin-6 (IL-6), thereby interfering with the IL-6/JAK2/STAT3 signaling pathway, an established inducer of DNMT1 expression (Dongoran et al., 2020).

For instance, statins have been shown to downregulate the RAS/PI3K/mTOR signaling cascade by the inhibiting the mevalonate pathway through DNA demethylation and the downregulation of the histone deacetylase HDAC2. This process begins with the inhibition of GTPase isoprenylation, leading to the reduced activity of RAS proteins and the MAPK pathway (Karlic et al., 2015). Another study found that statins were associated with the downregulation of DNMT 1, which may contribute to the overexpression of the cyclin-dependent kinase inhibitor p21, possibly reversing aberrant promoter p21 hypermethylation. However, this hypothesis has not been solved and remains to be conclusively demonstrated (Kodach et al., 2011; Dongoran et al., 2020).

In the context of CVDs, statins have also been associated with the upregulation of endothelial nitric oxide synthase (eNOS), which promotes vasodilatation, prevents thrombosis, and improves endothelial cell function in patients with hypertension and atherosclerosis (Chen et al., 2024). This effect may be partially mediated through epigenetic modulation.

Notably, statins have also been reported to modulate DNA hypermethylation in specific contexts. For instance, they can promote the overexpression of sirtuin 1 (SIRT1), which recruits DNMT 3B to CpG islands, leading to transcription repression of target genes (Zhang and Kraus, 2010; Allen and Mamotte, 2017). Furthermore, under high simvastatin doses, reduced acetylation of NF-κB has been observed, which suppresses its transcriptional activity and downregulates expression of pro-inflammatory genes such as TNF-α (Du et al., 2014).

Taken together, these findings highlight the regulatory capacity of statins on DNA methylation depending on the cellular context and target pathways. Further research on DNMT regulation by statins could provide further comprehension into novel epigenetic therapies aimed at modulating gene expression in pathological processes such as cancer or CVDs.

### Statins and histone modifications

Statins have been also involved in the regulation of epigenetic histone modifications, contributing to their broad spectrum of biological effects (Allen and Mamotte, 2017). Various studies have described that statins can influence gene expression through increased histone acetylation of histones H3 (Tikoo et al., 2015) and H4 (Singh et al., 2016). One proposed mechanism involves the inhibition of the mevalonate pathway, which leads to the intracellular accumulation of acetyl-CoA. This excess of acetyl-CoA may serve as a substrate for histone acetyltransferases, enhancing acetylation of the gene promoter regions and thereby promoting transcriptional activation (Allen and Mamotte, 2017; Cooney, 2010).

In addition to increasing acetyl-CoA availability, statins may also directly inhibit HDACs by binding to their active sites, suppressing their deacetylase activity (Singh et al., 2016; Kayzuka et al., 2025; Lin et al., 2008). This inhibition promotes histone acetylation, which neutralizes the positive charge on histones, allowing the loosening of chromatin structure. As a result, DNA becomes more accessible for the binding of transcription factors to promoter regions (Kayzuka et al., 2025; Allen and Mamotte, 2017; Bannister and Kouzarides, 2011).

Furthermore, statins have been correlated with the inhibition of histone methyltransferases (HMTs), potentially leading to the hypomethylation of histones and enhanced transcriptional activity (Kayzuka et al., 2025). These combined effects on histone acetylation and methylation suggest a significant role for statins in chromatin remodeling and gene regulation. Further molecular studies exploring how different types of statins influence these epigenetic processes could elucidate the underlying mechanisms of their protective roles in chronic diseases.

Several specific pathways have been described to illustrate these mechanisms. For example, statins downregulate the histone methyltransferase enhancer of zeste homolog 2 (EZH2), which in turn promotes the upregulation of HDAC5 and overexpression of the cyclin-dependent kinase inhibitor p27^KIP1^ (Ishikawa et al., 2014). Statins also downregulate HDAC activity, leading to increased histone-H3 acetylation at Sp1 binding sites within the p21 promoter (Lin et al., 2008). Moreover, inhibition of geranylgeranyl pyrophosphate (GGPP) synthesis by statins has been linked to the overexpression of p21, reinforcing their role in cell cycle regulation (Fuchs et al., 2008). Additionally, statins inhibit the GGTase–RhoA–YAP–SOX9 signaling axis, contributing to chromatin remodeling and further supporting their involvement in epigenetic regulation (Chen et al., 2024; Liu et al., 2023).

### Statins and microRNA regulation

MicroRNAs (miRNAs) are small non-coding RNAs, typically 18–25 nucleotides in length, that regulate gene expression at the post-transcriptional level (Yao et al., 2019). miRNAs can act as epigenetic modulators by targeting enzymes involved in chromatin remodeling and epigenetic modifications, such as DNMTs, HDACs, and HMTs (Yao et al., 2019). Conversely, miRNA expression is itself subject to regulation by epigenetic mechanisms. DNA methylation and histone modifications can modulate the expression of miRNAs, indicating a complex bidirectional interaction between miRNAs and epigenetic processes (Yao et al., 2019). Table 1 describes the miRNAs associated with statins role in functional significance and clinical relevance in various human diseases.

**TABLE 1:** Functional and clinical relevance of the miRNAs and its statin-induced expression.

miRNAs	Functional significance	Clinical relevance	Statin-induced expression
miR-129	May play role in apoptosis (Karaayvaz et al., 2013)	Role in heart disease, epilepsy, Alzheimer’s disease, obesity, diabetes, bone degeneration, osteosarcoma, nasopharyngeal carcinoma, and various other cancers (Deng et al., 2021)	Upregulated (Cerda et al., 2021)
miR-143	Promotes adipocyte differentiation by regulating extracellular signal-regulated kinase 5 (Liu et al., 2022)	Associated with adenomas, carcinomas, and colon, prostate and breast cancer (Gomes et al., 2016; Rasmi et al., 2023; Bolayırlı et al., 2022; Tokumaru et al., 2020)	Upregulated (Cerda et al., 2021)
miR-205	Acts in tissue morphogenesis and homeostasis and acts as tumor suppressor (Ferrari and Gandellini, 2025)	Altered expression in prostate, breast, lung, renal, head and neck endometrial, bladder cancer and melanoma (Ferrari and Gandellini, 2025)	Upregulated (Cerda et al., 2021)
miR-381	Functions as a tumor suppressor (Zeng et al., 2020)	Implicated in various cancers, including breast, endometrial, lung and other types (Zeng et al., 2020)	Upregulated (Cerda et al., 2021)
miR-495	Primarily functions as a tumor suppressor (Chen et al., 2017)	Associated with cancer and neurological disorders (Chen et al., 2017; Pang et al., 2024)	Upregulated (Cerda et al., 2021)
miR-29b	Regulates osteoblast development and tumor suppressor (Grassilli et al., 2022; Li et al., 2009)	Involved in cardiovascular disease and cancer (Grassilli et al., 2022; Liu et al., 2021)	Downregulated (Cerda et al., 2021)
miR-33a	Regulates fatty acid metabolism (Dávalos et al., 2011)	Linked to cancer and metabolic diseases (Dávalos et al., 2011; Weihua et al., 2020)	Downregulated (Cerda et al., 2021)
miR-17-5p	Essential for proliferation, cell cycle regulation and apoptosis (Stoen et al., 2021)	Altered expression in various cancer types (Stoen et al., 2021)	Downregulated (Zambrano et al., 2015), upregulated (Saavedra et al., 2022)
miR-20a-5p	Functions as tumor promoter and tumor suppressor (Tylden et al., 2024)	Associated with cancer progression and cholesterol regulation (Saavedra et al., 2022; Tylden et al., 2024)	Downregulated (Zambrano et al., 2015)
miR-106a-5p	Regulates angiogenesis and the activity of vascular endothelial and smooth muscle cells (Du et al., 2023)	Promotes several cancer types (Zhou et al., 2021)	Downregulated (Zambrano et al., 2015)
miR-483-5p	Maintains cellular function (Matson et al., 2023)	Linked to cancer and cardiovascular diseases (Zhao et al., 2023)	Upregulated (Lin et al., 2020)
miR-4667-5p	Limited functional data, Involved in skin photoaging (LUI et al., 2023)	Not well-characterized clinically	Upregulated (Lin et al., 2020)
miR-3609	Mediates proliferation and apoptosis (Ding et al., 2021)	Associated with cancer, glioma and other disorders (Ding et al., 2021)	Upregulated (Lin et al., 2020)
miR-1244	Involved in endoplasmic reticulum stress response (Czechowicz et al., 2024)	Not well-characterized clinically	Upregulated (Lin et al., 2020)

### Statins and lncRNA regulation

Long non-coding RNAs (lncRNAs) are transcripts of approximately 200 nucleotides that do not encode proteins. Despite being non protein-coding regions, lncRNAs play diverse and essential biological roles, including participation in chromosomal organization, telomere maintenance, and the structural organization of subcellular compartments. Notably, lncRNAs can also mediate epigenetic regulation by modulating chromatin structure, transcription, and post-transcriptional processes (Mercer et al., 2009).

Recent studies suggest that, in addition to their effect on miRNAs, statins also regulate lncRNAs, contributing to their pleiotropic actions (Tsilimigras et al., 2021). For example, the lncRNA *RP1-13D10.2*, has been shown to regulate LDLR expression and modulate the individual response to statin therapy (Mitchel et al., 2016). Similarly, another study identified LASER, a lncRNA involved in cholesterol homeostasis, may serve as a therapeutic target to enhance statins efficacy (Li et al., 2019). The lncRNA **MANTIS** has also been associated with statin-mediated vascular protection (Leisegang et al., 2019). Additionally, the lncRNA H19 has been implicated in the statin-mediated therapeutic response in patients with acute myocardial infarction (Huang et al., 2020).

In the context of atherosclerosis, statin were found to regulate pyroptosis-associated lncRNAs such as NEX-AS1 and NEXN, exerting protective effects that are independent of lipid-lowering activity (Wu et al., 2020). Furthermore *OLMALINC*, an oligodendrocyte maturation-associated long intergenic noncoding RNA, has been linked to the epigenetic regulation of genes involved in cholesterol biosynthesis, such as stearoyl-coenzyme A desaturase and shows strong associations with both statins use and serum triglycerides levels (Benhammou et al., 2019).

A more recent study reported that RP1-13D10.2, MANTIS, and lncHR1 were overexpressed in individuals with hypercholesterolemia, and that atorvastatin treatment significantly suppressed lncHR1 expression (Paez et al., 2023).

Collectively, these findings underscore the important role of lncRNAs in the epigenetic regulation mediated by statins. The identification of statin-responsive lncRNAs opens new avenues for personalized medicine and suggests novel molecular targets for improving the therapeutic efficacy of statins across a range of lipid-related and inflammatory diseases.

## Clinical evidence on statins regulating epigenetic modifications

### Cardiovascular diseases and atherosclerosis

Clinical evidence supports the protective role of statins in reducing cardiovascular risk, extending beyond their lipid-lowering effects. Emerging research highlights that epigenetic mechanisms contribute significantly to the pleiotropic benefits of statins. These compounds influence gene expression through modulation of DNA methylation, histone post-translational modifications, and non-coding RNAs, particularly in vascular endothelial cells (Kayzuka et al., 2025).

For instance, simvastatin has been shown to suppress the epigenetic activation of the YAP-SOX9 axis, thereby inhibiting endothelial-to-mesenchymal transition (EndMT)—a process implicated in vascular dysfunction and atherosclerosis progression (Liu et al., 2023). Similarly, atorvastatin has been reported to upregulate *SIRT1* expression at both the transcriptional and protein levels in patients with coronary artery disease, linking statin therapy to pathways associated with endothelial protection and cellular longevity (Tabuchi et al., 2012).

In experimental models of atherosclerosis, rosuvastatin enhances histone H3 and H4 acetylation by inhibiting HDAC6 and HDAC7, leading to increased expression of *CCR7*, a chemokine receptor involved in macrophage migration and plaque remodeling (Feig et al., 2011). This effect is mediated through SREBP-2-dependent displacement of HDAC6/7 from the CCR7 promoter, allowing recruitment of histone acetyltransferases (HATs) such as p300, therey promoting transcriptional activation via histone acetylation (Feig et al., 2011).

Statins also epigenetically regulate *PCSK9*, a key gene in cholesterol homeostasis and fatty acid metabolism. They increase PCSK9 expression through SREBP2 activation, which recruits cofactors like NPAT and TRRAP to facilitate histone H4 acetylation (Dong et al., 2010; Li and Liu, 2012). This chromatin remodeling enables the recruitment of HATs such as p300 and CBP, promoting active transcription via H3K9 acetylation and H3K4 trimethylation (Duddu et al., 2025). Although this upregulation of PCSK9 may reduce the lipid-lowering efficacy of statins, it uncovers a precise epigenetic mechanism that could be pharmacologically targeted.

In addition to chromatin remodeling, statins modulate miRNA expression, contributing to both lipid regulation and inflammation control (Ruiz-Pozo et al., 2023). In HepG2 cells, atorvastatin upregulates miR-129, miR-143, miR-205, miR-381, and miR-495, while downregulating miR-29b and miR-33a—miRNAs involved in lipogenesis and lipid metabolism (Cerda et al., 2021). Other studies have reported a decrease in hsa-miR-17-5p, hsa-miR-20a-5p, and hsa-miR-106a-5p with atorvastatin treatment (Zambrano et al., 2015), although contrasting findings suggest that miR-17-5p may also be upregulated and associated with LDLR suppression (Saavedra et al., 2022).

Furthermore statin treatment has been associated with the upregulation of miR-483-5p, miR-4667-5p, miR-3609, and miR-1244, all of which are implicated in the regulation of inflammatory responses. Notably, miR-483-5p may inhibit RhoA-mediated pathways, which are critical for monocyte migration and cytoskeletal dynamics. These miRNAs also appear to interact with the TGF-β signaling pathway, known for its dual role in immune modulation within atherosclerotic plaques (Lin et al., 2020).

Collectively, these findings underscore the role of statins as epidrugs—agents capable of modulating the epigenome—offering new avenues for therapeutic optimization in cardiovascular disease management.

### Cancer

Although statins are primarily recognized for their cholesterol-lowering effects, increasing evidence supports their potential as anticancer agents, particularly through the modulation of epigenetic mechanisms involved in tumorigenesis. These effects include alterations in DNA methylation, histone modifications, and non-coding RNA expression, which collectively influence gene regulation, cell cycle progression, and tumor cell differentiation (Awosika et al., 2023; Kayzuka et al., 2025; Mohammadzadeh et al., 2020).

In oral squamous cell carcinoma (OSCC), cerivastatin and simvastatin have been shown to significantly suppress DNMT1, a key enzyme responsible for maintaining promoter hypermethylation of tumor suppressor genes. This suppression leads to reactivation of genes such as p21, resulting in G0/G1 cell cycle arrest and reduced tumor proliferation (Dongoran et al., 2020). Given the frequent overexpression of DNMT1 in various malignancies, these findings highlight a promising epigenetic mechanism for statin-mediated tumor suppression.

In a broader oncological context, simvastatin and ibandronate have been shown to modulate the mevalonate pathway in breast, prostate, and osteosarcoma cell lines. This inhibition reduces the isoprenylation of small GTPases like RAS, leading to downregulation of DNMT1, HDACs, and specific miRNAs. These epigenetic changes promote the demethylation and activation of pro-apoptotic and differentiation-related genes. Notably, simvastatin significantly upregulates miR-612, a miRNA associated with reduced tumor cell pluripotency and enhanced sensitivity to 5-fluorouracil, suggesting a potential suggesting a potential chemosensitizing role for statins (Karlic et al., 2015).

In colorectal cancer (CRC), statins such as simvastatin, fluvastatin, and atorvastatin exert epigenetic effects that are independent of the mevalonate pathway. These include inhibition of EZH2, a HMT that represses tumor suppressor genes. EZH2 inhibition leads to upregulation of p27^KIP1^, promoting cellular differentiation and improved patient survival. Furthermore, combining statins with class II HDAC inhibitors has been shown to enhance these anticancer effects synergistically (Ishikawa et al., 2014).

Another mechanism involves lovastatin, which promotes demethylation of the *BMP2* gene, encouraging tumor differentiation and reducing aggressiveness. This protein is part of the Bone Morphogenetic Proteins (BMPs) family involved in intestinal epithelial cell differentiation, inhibition of stem cell activity, and maintenance of adult tissue homeostasis. DNMT inhibition facilitates *BMP2* demethylation and upregulation in CRC cells, sensitizing tumors to chemotherapeutic agents. While additional studies are required to validate its efficacy and define its clinical application, these findings underscore the potential of statins as adjuvant epigenetic agents (Wang et al., 2014).

Despite these promising findings, some studies have reported inconsistent results, with no significant changes in histone acetylation and, in some cases, increased DNMT activity following statin treatment (Bridgeman et al., 2019). These discrepancies underscore the complexity of statin-epigenome interactions and the need for further mechanistic and translational research.

In summary, statins are emerging as multifunctional agents with potential applications in oncology, particularly as adjuvant modulators of the epigenome. Their ability to influence chromatin remodeling, gene expression, and non-coding RNA networks supports their integration into personalized cancer therapies, pending further validation in preclinical and clinical settings.

### Diabetes and insulin resistance

Growing evidence suggests a paradoxical association between statin therapy and an increased risk of type 2 diabetes mellitus (T2DM), primarily through mechanisms that promote insulin resistance (Paseban et al., 2019). T2DM is a multifactorial disease influenced by genetic predisposition, environmental exposures, and pharmacological interventions (Beulens et al., 2021). Among these, epigenetic mechanisms have emerged as critical contributors to the pathogenesis of insulin resistance and impaired glucose metabolism (Awosika et al., 2023).

An important study involving approximately 4,760 participants from the Framingham Heart Study Offspring cohort (FHS) and the Women’s Health Initiative (WHI) identified a specific epigenetic marker associated with statin use. DNA methylation at CpG site cg06500161 within the *ABCG1* gene was positively correlated with statin therapy, elevated fasting glucose, increased insulin levels, and a higher risk of T2DM (Qie et al., 2021). Given ABCG1’s dual role in cholesterol efflux and glucose homeostasis, this finding underscores the gene’s central role in the metabolic interplay between lipid and glucose regulation (Kotlyarov and Kotlyarova, 2025; Liu et al., 2020).

Further supporting this, a comparative epigenome-wide association study in statin-treated versus non-treated T2DM patients identified 79 differentially methylated CpG sites, with three—cg17901584 (*DHCR24*), cg27243685 (*ABCG1*), and cg05119988 (*SC4MOL*)—showing strong associations with statin exposure (Schrader et al., 2021). While *DHCR24* and *SC4MOL* are primarily involved in cholesterol biosynthesis, methylation at *DHCR24* was also linked to glucose metabolism, suggesting a shared epigenetic axis between lipid and glycemic pathways (Peeples et al., 2024).

In addition to DNA methylation, miRNA dysregulation has also been implicated in statin-induced metabolic changes. For instance, rosuvastatin has been shown to deregulate miR-27a and miR-221, both of which are involved in insulin signaling and glucose uptake (Serik et al., 2021). Simvastatin dose-dependently increases miR-27a expression in hepatic cells, which indirectly reduces LDL receptor (LDLR) levels by upregulating PCSK9, a protein that promotes LDLR degradation. Under hyperglycemic conditions, this dysregulation may impair lipid clearance and exacerbate insulin resistance (Galicia-Garcia et al., 2020).

Moreover, miR-33a and miR-33b, which regulate *ABCA1* and *ABCG1*, are reportedly overexpressed in statin users. These genes are essential for pancreatic beta-cell function, and their suppression may impair insulin secretion and glucose regulation (Awosika et al., 2023). Clinical studies have corroborated these molecular findings. A 10-week trial of high-intensity atorvastatin therapy demonstrated increased insulin resistance and compensatory insulin secretion, suggesting a shift toward glucose intolerance in susceptible individuals (Abbasi et al., 2021). A systematic review further confirmed that statin use is associated with reduced insulin sensitivity and increased insulin resistance, raising concerns for patients at risk of developing diabetes (Dabhi et al., 2023).

Collectively, these findings highlight the epigenetic complexity underlying statin-induced metabolic effects. Through DNA methylation and miRNA modulation, statins may inadvertently disrupt glucose homeostasis. These results emphasize the need for personalized risk assessment and epigenetic monitoring in patients undergoing long-term statin therapy.

### Other diseases

Conversely, statins exhibit notable immunomodulatory and anti-inflammatory properties, making them promising candidates for the treatment of autoimmune diseases. Statins can regulate immune responses through both mevalonate pathway-dependent and -independent mechanisms, affecting antigen-presenting cells and T-cell functions (Dehnavi et al., 2020). Evidence has demonstrated improvements in conditions such as rheumatoid arthritis, lupus, and multiple sclerosis, including enhancements in cytokine profiles and clinical markers like C-reactive protein and erythrocyte sedimentation rate (ESR). However, the precise mechanisms and optimal doses required for these immunomodulatory effects remain unclear (Dehnavi et al., 2020).

Furthermore, statins have demonstrated significant neuroprotective effects, potentially reducing the incidence of neurodegenerative diseases. For instance, a large retrospective cohort study involving 288,515 participants found that statin use is associated with a substantial reduction in the risk of various neurodegenerative diseases, including Alzheimer’s disease, dementia, multiple sclerosis, Parkinson’s disease, and amyotrophic lateral sclerosis (Torrandell-Haro et al., 2020). These findings have fueled interest in drug repurposing, as the anti-inflammatory and antioxidant properties of statins may help reduce amyloid plaque formation and protein aggregation, both central to the pathogenesis of Alzheimer’s and Parkinson’s diseases (Bhat et al., 2020). Moreover, a meta-analysis of 55 observational studies encompassing over seven million patients revealed that prolonged statin exposure (more than 3 years) significantly enhances dementia risk reduction, with rosuvastatin displaying the most pronounced protective effects (Westphal Filho et al., 2025).

Emerging evidence also suggests that statins may delay cellular aging and combat senescence. These agents have been shown to improve cellular function, mitigate telomere shortening, reduce apoptosis, and counteract the senescence-associated secretory phenotype (SASP) (Bahrami et al., 2020; Guaraldi et al., 2023; Strazhesko et al., 2016). Together, these findings illustrate the broader physiological impacts of statins, highlighting their potential benefits beyond lipid regulation while also underscoring the need for careful evaluation of long-term safety.

## Limitations and future perspectives

Together, these findings illustrate the broader physiological impacts of statins, highlighting their potential benefits beyond lipid regulation while also underscoring the need for careful evaluation of long-term safety (Awosika et al., 2023). Advancing the understanding of statin-induced epigenetic modifications could expand their therapeutic applications beyond cardiovascular disease, potentially informing the management of cancer, neurodegenerative disorders, autoimmune diseases, and metabolic dysfunctions. To fully harness this potential, healthcare providers and researchers must recognize the epigenetic dimensions of statin action and support research strategies that prioritize both mechanistic depth and clinical relevance.

## Conclusion

In conclusion, while statins are primarily prescribed for the management of LDL cholesterol, increasing evidence supports their role as modulators of the epigenome. Their capacity to influence DNA methylation, histone modifications, and miRNA expression implicates statins in the regulation of key biological processes, including inflammation, endothelial function, and tumor suppression.

Preclinical and clinical studies have demonstrated that these epigenetic mechanisms may underlie the beneficial effects of statins across a range of conditions, including cardiovascular disease, cancer, and diabetes. However, statins have also been linked to adverse metabolic effects, such as increased insulin resistance and heightened risk of T2DM. Therefore, it is essential to further investigate the molecular pathways involved in statin-mediated disease modulation.

Large-scale, longitudinal studies incorporating epigenomic profiling and integrative molecular analyses are needed to more precisely define the benefits and risks of statin therapy. Such efforts will be critical to developing personalized therapeutic strategies that optimize statin efficacy while minimizing unintended effects.
